# From the muscle hypothesis to a muscle solution?

**DOI:** 10.1002/ehf2.12423

**Published:** 2019-02-23

**Authors:** Andrew J. Stewart Coats

**Affiliations:** ^1^ IRCCS, San Raffaele Pisana Rome Italy

In this issue, ESC Heart Failure publishes an extremely interesting paper.^1^ Sabbah and colleagues tested elamipretide (ELAM), a peptide that targets mitochondria, thereby possibly affecting many organs, but clearly with skeletal muscle also a key target. In their well‐established and characterized canine model of chronic intracoronary microembolization‐induced heart failure (HF), 3 months of ELAM therapy led to a shift in type 1 to type 2 fibres, restoring the balance towards normal. In addition, skeletal muscle mitochondrial function in the HF dogs was abnormal in terms of ADP‐dependent mitochondrial respiration, membrane potential, permeability transition pore, stimulated ATP synthesis, and cytochrome *c* oxidase activity; and all of these were near normalized by short‐term *in vitro* application of ELAM. Sabbah and colleagues conclude that ‘ELAM, previously shown to positively influence mitochondrial function of the failing heart, can also positively impact mitochondrial function of skeletal muscle and potentially help restore skeletal muscle function and improve exercise tolerance’. This is a big claim and one that if true in the human HF condition could potentially be a significant treatment advance for HF, and plausibly other chronic disorders.

Way back in the 1980s, my group and that of several others concentrated on skeletal muscle changes in the patients with chronic treated congestive HF in an attempt to explain the persistently poor exercise tolerance, which correlated extremely weakly, if at all, with global measures of central haemodynamic reserve. We all came to the conclusion that skeletal muscle was a stronger candidate for being the weak link in the exercise process than short‐term cardiovascular reserve in determining the exercise tolerance of the patient with non‐oedematous HF. Much of this work has been summarized in two of our early papers, highlighting the central role of peripheral changes^2^ and coining the term ‘muscle hypothesis’^3^, ^4^ to explain symptoms and even aspects of disease progression in congestive HF. A derivative thought from this hypothesis was that effective treatments for the abnormal muscle of HF may improve exercise tolerance and even disease progression, yet treatment development for HF to date has largely remained focused on seeking cardio‐active treatments. Exercise training, which has a central role in the treatment of stable HF, does so in all probability via beneficial changes on skeletal muscle rather than via direct cardiovascular or haemodynamic effects.^5^ No other HF treatment with this skeletal muscle mode of action has emerged, perhaps until now. It is a long way to go from animal model experiments showing *in vitro* and *in vivo* effects of putative benefit to a successful clinical trial, yet the glimmer of hope of a novel mode of action addressing a major part of the pathophysiology of human HF^6^, ^7^, ^8^ remains tantalizing. If we can improve the failing muscle, we could potentially improve gross muscle function, reduce fatigability, and also, via reducing the action of the overactive muscle ergo‐reflex, potentially reduce sympathetic drive and sympatho‐vagal imbalance. As an aside, similar muscle changes as are seen in HF^9^, ^10^, ^11^ are also seen in many chronic conditions associated with the risk of cachexia and sarcopenia. If these results hold true for HF, they may also show potential for renal,^12^, ^13^ liver,^14^, ^15^, ^16^ cancer,^17^, ^18^, ^19^, ^20^, ^21^, ^22^, ^23^ and other chronic cachexias^24^, ^25^ and their therapies,^26^ thus introducing for the first time a treatment that crosses traditional discipline boundaries more effectively than any to date.
